# Poor prognosis of hexokinase 2 overexpression in solid tumors of digestive system: a meta-analysis

**DOI:** 10.18632/oncotarget.15974

**Published:** 2017-03-07

**Authors:** Jiayuan Wu, Liren Hu, Fenping Hu, Lei Zou, Taiping He

**Affiliations:** ^1^ Clinical Research Center, The Affiliated Hospital of Guangdong Medical University, Zhanjiang, Peoples Republic of China; ^2^ Nutritional Department, The Affiliated Hospital of Guangdong Medical University, Zhanjiang, Peoples Republic of China; ^3^ Department of Epidemiology and Health Statistics, School of Public Health, Guangdong Medical University, Zhanjiang, Peoples Republic of China; ^4^ Department of Radiotherapy, The Seventh Peoples Hospital of Chengdu, The Oncology Hospital of Chengdu, Chengdu, Peoples Republic of China; ^5^ Department of Hepatobiliary Surgery, The First Peoples Hospital of Yunnan Province, Kunming, Peoples Republic of China; ^6^ School of Public Health, Guangdong Medical University, Zhanjiang, Peoples Republic of China

**Keywords:** hexokinase 2, solid tumors, digestive system, prognosis, meta-analysis

## Abstract

Several previous studies have reported the prognostic value of hexokinase 2 (HK2) in digestive system tumors. However, these studies were limited by the small sample sizes and the results were inconsistent among them. Therefore, we conducted a meta-analysis based on 15 studies with 1932 patients to assess the relationship between HK2 overexpression and overall survival (OS) of digestive system malignancies. The relationship of HK2 and clinicopathological features was also evaluated. Hazard ratio (HR) or odds ratio (OR) with its 95% confidence intervals (CI) were calculated to estimate the effect size. Positive HK2 expression showed poor OS in all tumor types (HR = 1.75 [1.41-2.18], *P* < 0.001). When stratified by tumor type, the impact of HK2 overexpression on poor prognosis was observed in gastric cancer (HR = 1.77 [1.25-2.50], *P* < 0.001), hepatocellular carcinoma (HR = 1.87 [1.58-2.21], *P* < 0.001), and colorectal cancer (HR = 2.89 [1.62-5.15], *P* < 0.001), but not in pancreatic ductal adencarcinoma (HR = 1.11 [0.58-2.11], *P* = 0.763). Furthermore, high HK2 expression was significantly associated with some phenotypes of tumor aggressiveness, such as large tumor size (OR = 2.03 [1.10-3.74], *P* = 0.024), positive lymph node metastasis (OR = 2.05 [1.39-3.02], *P* < 0.001), advanced clinical stage (OR = 2.17 [1.21-3.89], *P* = 0.009) and high alpha fetoprotein level (OR = 1.47 [1.09-2.02] *P* = 0.013). In summary, HK2 might act as a prognostic indicator and a potential therapeutic target of these digestive system cancers.

## INTRODUCTION

The digestive system is composed of many ducts and glands, and because of its complicated physiology and anatomy, numerous diseases are prone to occur, especially malignancies, which have become one of the most terrible threats for human health [1]. According to the 2015 cancer statistics, the cancer-related mortality has been continuously declining for the past two decades. However, the morbidity of some digestive system malignancies, including cancers of the esophagus, intestine, liver and pancreas, is still increasing [2]. Cancers originated from some parts of the digestive system, such as stomach, colon, rectum, liver, pancreas, and esophagus, are highly prevalent, and rank among the top causes of cancer-related death worldwide. Although plenty of biomarkers have been identified in digestive system malignancies, the prognosis remains to be dismal because of the high incidence of local recurrence, lymph node invasion and distant metastasis [3]. Moreover, patients with the same tumor characteristics, for example clinical stage, tumor differentiation and tumor size, may suffer from diverse clinical outcomes [4]. Therefore, it is necessary to identify a new reliable marker to obtain additional prognostic information, and supply more effective therapies to avoid patients succumbing to these digestive neoplasms.

Reprogramming energy metabolism is one of the key hallmarks of many rapidly growing cancers [5]. With regard to the Warburg effect, cancer cells favor a metabolic shift towards anaerobic glycolysis even in the presence of sufficient oxygen [6, 7]. Because the tumor microenvironment is characterized by hypoxia, increased glycolysis provides tumor cells with rapid energy production to obtain a survival advantage [8]. While oxidative phosphorylation (OXPHOS) metabolize glucose almost exclusively for maximal ATP generation, glycolytis breakdown of glucose produces various intermediate metabolites to satisfy the anabolic need of the rapidly divided cancer cells, including provision of intermediates for growth, production of excess lactate that promotes tumor invasion, and adaptation to unfavorable microenvironmental conditions like hypoxia or chemotherapy [9, 10]. Moreover, both free radicals and reactive oxygen species (ROS) raised by OXPHOS could break double-stranded DNA leading to cell death, thus low level of OXPHOS may reduce the production of the abovementioned apoptotic cytokines and help cancer cells to escape from the cytotoxic effects of oxidative damage [11]. Therefore, the Warburg effect itself involves high level of aerobic glycolysis catalyzed by pivotal enzymes offers several advantages for tumorigenesis, which can serve as a potential mechanism for targeting glucose metabolism as a therapeutic approach in cancer treatment.

Hexokinase (HK) catalyzes the conversion of glucose to glucose-6-phoshate (G6P), and is the first and rate-limiting step of both anaerobic glycolysis and ultimately oxidative phosphrylation. The four members of the HK family (HK1-4) in mammals are structurally similar but expressed in a tissue-specific manner [12]. Among them four HK enzymes, HK2 is rarely expressed in normal tissues, except some insulin-sensitive tissues, such as skeletal, adipose and cardiac muscle [13]. By contrary, HK2 is observed to be high expressed in several types of tumor cell, indicating that it plays a critical role in tumor initiation and development [14, 15]. Immunolocalization of HK2 protein has been reported in several human carcinomas of digestive system, and may be a promising prognostic biomarker for them [16–30]. However, due to the inconsistency of the results, the prognostic value of HK2 in digestive system tumors is still inconclusive, and needs to be confirmed by systematic analyses. Therefore, we conducted this meta-analysis to more precisely estimate the relationship between HK2 overexpression and its prognostic value in solid tumors of digestive system by reviewing all relevant studies.

## RESULTS

### Description of the included studies

**Figure 1:**
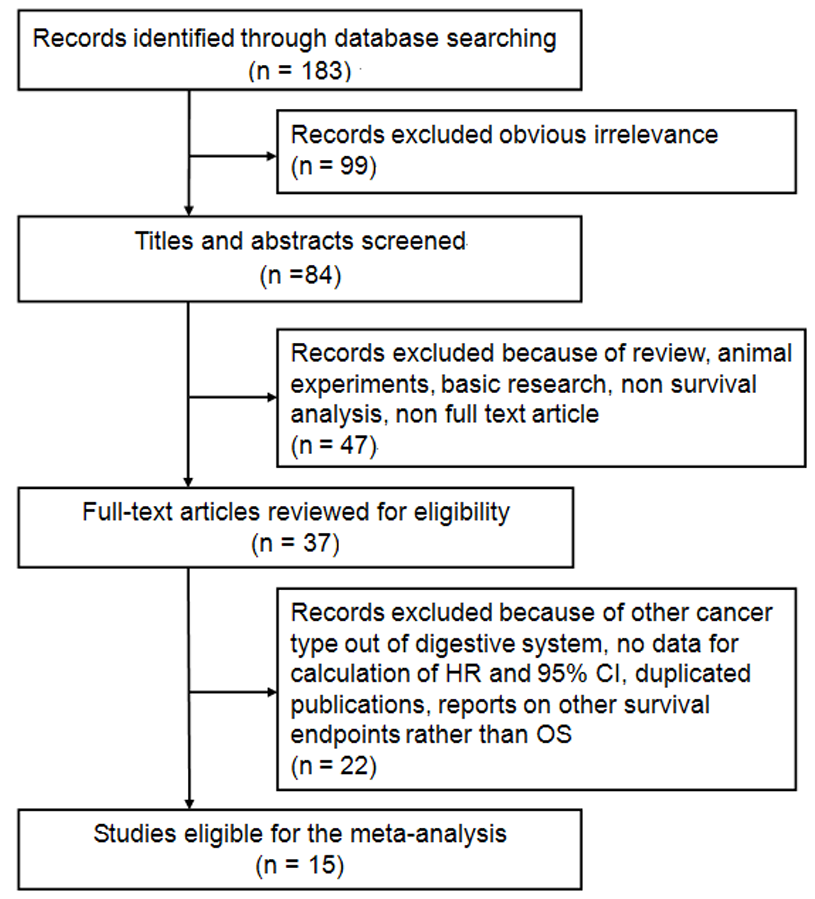
Flow diagram of the study selection process and specific reasons for exclusion in the meta-analysis 183 studies were preretrieved in accordance with the established search strategies. Of these articles, 99 were excluded because of clear lack of relevance. The remaining 84 studies were further screened out through browsing the titles and abstracts, and then 47 were removed based on the eligible criteria. After reading the full texts of 37 studies, 15 eligible studies were finally included in this meta-analysis.

The concise process of literature selection was shown in Figure 1. Initially, 183 papers were generated in the primary electronic search in major databases. According to the inclusion criteria, 15 eligible studies [16–30] published from 2007 to 2016 were included. A total of 1932 patients from China [16, 21, 22, 25, 26], Japan [17, 20, 28, 30], Korea [23, 29], United States of America [18, 24], Canada [19], and Taiwan [27] were diagnosed with various cancers, including hepatocellular carcinoma (HCC) [16, 21, 22, 24, 26, 27, 28], pancreatic ductal adenocarcinoma (PDAC) [18, 20, 30], gastric cancer (GC) [23, 25, 29], and colorectal cancer (CRC) [17, 19]. Among these 15 included studies, HK2 expression was evaluated by immunohistochemistry (IHC) method in 12 studies, by reverse transcription-polymerase (RT-PCR) in 2 cohorts [22, 27], and by immunofluorescence (IF) method in only one research [19]. Only six studies had performed blinded reading during evaluating HK2 expression [18, 19, 23–25, 28]. The median follow-up time for all included studies ranged from 23 to 60 months, even 5 studies did not report it [18, 20, 23, 28, 30]. Of the 15 articles, cutoff value for defining positive HK2 expression could be retrieved from 12 original studies. The hazard ration (HR) estimates and the corresponding confidence intervals (CIs) in 8 studies were directly extracted through multivariate analyses and those of 7 other cohorts were calculated from univariate analysis or Kaplan-Meier survival curves [18, 19, 21–23, 27, 30]. According to the quality criteria, all cohort studies were of high quality and had scores of 6 or more. The main characteristics of the included studies were listed in Table 1 and the complete process of literature search was showed in Figure S1.

**Table 1:** Main characteristics of 15 eligible studies in the meta-analysis

Study (authors-year)	Study region	Recruitment time	Sample size	Cancer type	Detection method	Follow up period	Blinding status	Cutoff scores (High/Low)	Analysis method	HR estimation	Quality score
Zhang ZF (2016) [16]	China	2000-2013	155	HCC	IHC	Up to 2014.3	NR	Score ≥ 5 * 66/89	Multivariate	OS: 2.70(1.76-4.15)	8
Katagiri M (2016) [17]	Japan	2000-2008	195	CRC	IHC	Median 58 (1-131)	NR	> 10% # 100/95	Multivariate	OS: 2.70(1.40-5.60)	8
Anderson M (2016) [18]	USA	NR	125	PDAC	IHC	NR	Yes	Score ≥ 4 60/65	Univariate	OS: 1.27(1.04-1.55)	7
Ho N (2016) [19]	Canada	2005-2011	60	CRC	IF	Up to 2013.5	Yes	F score ≥ 24.7 30/30	Univariate	OS: 3.37(1.17-9.75)	7
Ogawa H (2015) [20]	Japan	2007-2012	36	PDAC	IHC	NR	NR	Score ≥ 5 21/15	Multivariate	OS: 2.57(0.89-8.39)	6
Li YQ (2015) [21]	China	2006-2008	80	HCC	IHC	Median 30 (0-60)	NR	Score ≥ 2 56/24	Univariate	OS: 2.11(1.55-3.84) a	8
Guo WJ (2015) [22]	China	NR	120	HCC	RT-PCR	Up to 2014.12	NR	NR 72/48	Univariate	OS: 1.96 (1.36-2.82)	8
Hur H (2013) [23]	Korea	2006-2007	152	GC	IHC	NR	Yes	Score ≥ 2 7/145	Univariate	OS: 1.64(0.77-3.50) a	7
Kwee SA (2012) [24]	USA	1986-2009	159	HCC	IHC	Mean 48 (0-294)	Yes	Score ≥ 2 74/85	Multivariate	OS: 1.62(1.00-2.60)	9
Qiu MZ (2011) [25]	China	1999-2001	188	GC	IHC	Median 60 (3-120)	Yes	NR 40/148	Multivariate	OS: 3.48(1.56-7.72)	8
Gong L (2011) [26]	China	NR	97	HCC	IHC	Median 23.6 (1-50)	NR	> 10% 54/43	Multivariate	OS: 2.05(1.02-4.11)	8
Peng SY (2008) [27]	Taiwan	1982-1997	203	HCC	RT-PCR	> 5 years or until death	NR	NR 70/133	Univariate	OS: 1.50 (1.12-2.01)	7
Paudyal B (2008) [28]	Japan	1999-2007	31	HCC	IHC	NR	Yes	Score > 0 25/6	Multivariate	OS: 2.15(0.31-14.53)	8
Rho M (2007) [29]	Korea	1995-1995	257	GC	IHC	Mean 50 (1-72)	NR	> 10% 43/214	Multivariate	OS: 1.47(0.94-2.29)	7
Lyshchik A (2007) [30]	Japan	NR	74	PDAC	IHC	NR	NR	Score ≥ 3 38/36	Univariate	OS: 0.60 (0.37-0.96)	6

* different scores with combination of percentage of positives cells and intensity.

^#^ the proportion of the staining tumor cells by visual analysis of the immunohistochemistry.

^a^ extrapolated from survival curve. *OS* overall survival, *NR* data were not reported, *IHC* immunohistochemistry, *IF* immunofluorescence, *RT-PCR* reverse transcription-polymerase chain reaction, *HCC* hepatocellular carcinoma, *CRC* colorectal cancer, *PDAC* pancreatic ductal adenocarcinoma, *GC* gastric cancer.

### Correlation between HK2 expression and overall survival (OS)

**Figure 2:**
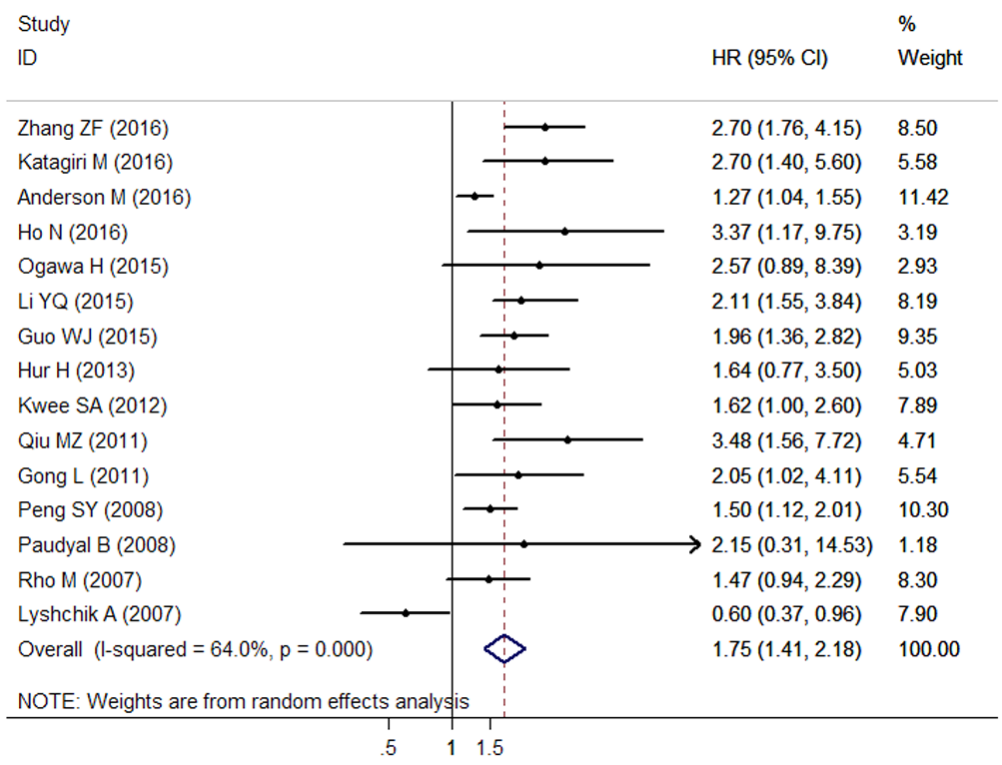
Forest plots of the overall outcome for overall survival (OS) in solid tumors of digestive system Hazard ratios (HRs) for each trial are represented by the squares, and the horizontal lines crossing the square stand for the 95% confidence intervals (CIs). The diamonds represent the estimated pooled effect of the overall outcome for OS in all solid tumors of digestive system. All *P* values are two-sided.

The combined analysis of 15 datasets showed a significant association between patients with HK2 overexpression and poor OS (pooled HR = 1.75; 95% CI = 1.41–2.18; *P* < 0.001; random effects) (Table 2; Figure 2). When the subgroup analysis was conducted by cancer type, the overall results revealed that high HK2 level significantly lead to the poor OS in patients with GC (pooled HR = 1.77; 95% CI = 1.25–2.50; *P* < 0.001; fixed effects), HCC (pooled HR = 1.87; 95% CI = 1.58–2.21; *P* < 0.001; fixed effects), and CRC (pooled HR = 2.89; 95% CI = 1.62–5.15; *P* < 0.001; fixed effects), but not in patients with PDAC (pooled HR = 1.11; 95% CI = 0.58–2.11; *P* = 0.763; random effects) (Table 2). The stratification according to detection method demonstrated that high HK2 expression was still an unfavorable predictor of OS in immunohistochemistry (IHC) detection (pooled HR = 1.74; 95% CI = 1.32–2.30; *P* < 0.001; random effects), in reverse transcription-polymerase chain reaction (RT-PCR) detection (pooled HR = 1.67; 95% CI = 1.33–2.09; *P* < 0.001; fixed effects), and in immunofluorescence (IF) detection (pooled HR = 3.37; 95% CI = 1.17–9.73; *P* = 0.025; random effects). Furthermore, this association did not only exist in the Eastern Asian population (pooled HR = 1.82; 95% CI = 1.39–2.38; *P* < 0.001; random effects), but also in the North American population (pooled HR = 1.35; 95% CI = 1.13–1.62; *P* = 0.001; fixed effects) (Table 2). Moreover, the results did not change when the analysis method, sample size, and blinding status were included (Table 2).

**Table 2:** Meta-analysis of HK2 overexpression and prognosis in digestive system cancers

Categories	Studies (patients)	HR (95% CI)	*I*^2^ (%)	*P*^h^	Z	*P*
Overall survival	15 (1932)	1.75 (1.41-2.18)	64.0%	< 0.001	5.02	< 0.001
Cancer type						
GC	3 (597)	1.77 (1.25-2.50) ^F^	42.1%	0.178	3.23	< 0.001
HCC	7 (845)	1.87 (1.58-2.21) ^F^	0.0%	0.449	7.37	< 0.001
PDAC	3 (235)	1.11 (0.58-2.11)	80.1%	0.007	0.30	0.763
CRC	2 (255)	2.89 (1.62-5.15) ^F^	0.0%	0.732	3.58	< 0.001
Detection method						
IHC	12 (1549)	1.74 (1.32-2.30)	68.7%	< 0.001	3.90	< 0.001
RT-PCR	2 (323)	1.67 (1.33-2.09) ^F^	20.5%	0.262	4.38	< 0.001
IF	1 (60)	3.37 (1.17-9.73)	NA	NA	2.25	0.025
Analysis method						
Multivariate	8 (1118)	2.09 (1.68-2.59) ^F^	1.2%	0.420	6.70	< 0.001
Univariate	7 (814)	1.48 (1.10-2.00)	73.3%	0.001	2.55	0.011
Sample size						
≥ 100	9 (1554)	1.78 (1.44-2.20)	56.1%	0.020	5.29	< 0.001
< 100	6 (378)	1.45 (1.10-1.90)	75.4%	0.001	2.63	0.008
Blinding status						
Yes	6 (715)	1.43 (1.21-1.70) ^F^	45.6%	0.102	4.11	< 0.001
NR	9 (1217)	1.74 (1.29-2.35)	71.3%	0.001	3.60	< 0.001
Study region						
Eastern Asia	12 (1588)	1.82 (1.39-2.38)	64.5%	0.001	4.35	< 0.001
North America	3 (344)	1.35 (1.13-1.62) ^F^	47.1%	0.151	3.27	0.001

All pooled *HR*s were derived from random-effect model except for cells marked with (fixed ^F^). *P*_h_ *P*-value for heterogeneity based on Q test. *P* *P*-value for statistical significance based on Z test. *NA* none available, *NR* none reported, *GC* gastric cancer, *HCC* hepatocelluar carcinoma, *PDAC* pancreatic ductal adencarcinoma, *CRC* colorectal cancer, *IHC* immunohistochemistry, *RT-PCR* reverse transcription-polymerase, *IF* immunofluorescence.

We analyzed the heterogeneity of the included datasets based on the *P* value for heterogeneity. Table 2 illustrates that all of the included datasets of OS had extreme heterogeneity (*I*^2^ = 64.0%, *P*_h_ < 0.001). Thus, we used a random-effects model to estimate the overall HR for OS. When the subgroup analysis was conducted to assess the source of heterogeneity based on cancer type, detection method, analysis method, study region, blinding status, and sample size, the heterogeneity was obvious to be still significantly evident (Table 2).

### Correlation between TSR and clinicopathological features

The relationship of HK2 expression with clinicopathological features are illustrated in Table 3. Positive HK2 expression was correlated with certain phenotypes of tumor aggressiveness, such as large tumor size (pooled odd ration [OR] = 2.03; 95% CI = 1.10–3.74; *P* = 0.024; random effects), positive lymph node metastasis (pooled OR = 2.05; 95% CI = 1.39–3.02; *P* < 0.001; fixed effects), advanced clinical stage (pooled OR = 2.17; 95% CI = 1.21–3.89; *P* = 0.009; random effects) and high alpha fetoprotein (AFP) level (pooled OR = 1.47; 95% CI = 1.09–2.02; *P* = 0.013; fixed effects). This finding indicated that HK2 may promote tumor invasion and aggressiveness. However, no association existed between HK2 expression and certain factors, such as gender (pooled OR = 0.82; 95% CI = 0.61–1.10; *P* = 0.185; fixed effects), depth of invasion (pooled OR = 2.00; 95% CI = 0.77–5.18; *P* = 0.152; random effects), differentiation (pooled OR = 1.16; 95% CI = 0.51–2.65; *P* = 0.728; random effects), distant metastasis (pooled OR = 1.99; 95% CI = 0.59–6.68; *P* = 0.265; random effects), HBV infection (pooled OR = 1.03; 95% CI = 0.57–1.86; *P* = 0.927; fixed effects), liver cirrhosis (pooled OR = 0.95; 95% CI = 0.61–1.48; P = 0.805; fixed effects), and portal vein involvement (pooled OR = 1.36; 95% CI = 0.49–3.74; *P* = 0.555; fixed effects).

**Table 3:** Meta-analysis of HK2 positive expression and clinicopathological features in digestive system cancers

Categories	Studies (patients)	OR (95% CI)	*I*^2^ (%)	*P*^h^	Z	*P*
Gender (male vs. female)	9 (1331)	0.87 (0.67-1.13) ^F^	0.0%	0.974	1.03	0.301
Tumor size (≥ 5 cm vs. < 5 cm)	8 (1140)	2.03 (1.10-3.74)	76.6%	< 0.001	2.26	0.024
Depth of invasion (T3+T4 vs. T1+T2)	6 (906)	2.00 (0.77-5.18)	82.5%	< 0.001	1.43	0.152
Lymph node metastasis (positive vs. negative)	5 (819)	2.05 (1.39-3.02) ^F^	0.0%	0.867	3.63	< 0.001
Clinical stage (III+IV vs. I+II)	7 (982)	2.70 (1.65-4.42)	57.7%	0.028	3.96	< 0.001
Differentiation (poor vs. well + moderate)	8 (1338)	1.56 (0.93-2.61)	63.6%	0.005	1.69	0.090
Distant metastasis (yes vs. no)	3 (532)	1.99 (0.59-6.68)	55.5%	0.106	1.11	0.265
AFP level (> 20 vs. ≤ 20 ng/ml)	6 (725)	1.48 (1.09-2.02) ^F^	0.0%	0.667	2.49	0.013
HBV infection (yes vs. no)	5 (655)	0.78 (0.52-1.18) ^F^	0.0%	0.820	1.19	0.235
Liver cirrhosis (yes vs. no)	3 (402)	0.95 (0.61-1.48)	2.0%	0.361	0.25	0.805
Portal vein involvement (postive vs. negative)	2 (116)	1.36 (0.49-3.74) ^F^	0.0%	0.980	0.59	0.555

All pooled *ORs* were derived from random-effect model except for cells marked with (fixed ^F^). *P*_h_ *P*-value for heterogeneity based on *Q* test.

*P P*-value for statistical significance based on *Z* test. *OR* odd ratio; *HBV* hepatitis B virus; *AFP* alpha fetoprotein.

### Cumulative meta-analysis and meta-regression analysis

**Figure 3:**
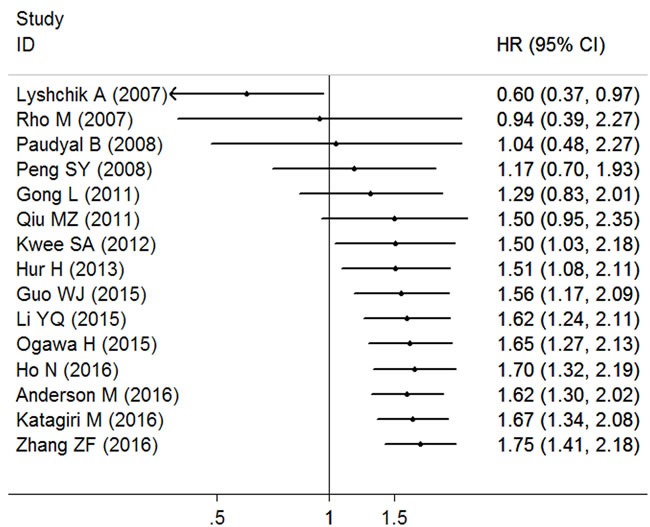
Cumulative meta-analysis of HK2 expression and OS in solid tumors of digestive system The changes of the pooled hazard ratios (HRs) over time are represented by the squares, and the horizontal lines crossing the square stand for the coresponding 95% confidence intervals (CIs).

A cumulative meta-analysis of 15 cohorts was performed to evaluate the cumulative HR estimate over time. The results of cumulative meta-analysis are shown in Figure 3. The following can be summarized: 1) high HK2 expression was regarded as a protective factor of prognosis according to the publication by Lyshchik A in 2007; 2) after including more literatures, HK2 overexpression had changed to be a significant influencing factor of unfavorable survival; 2) after including some studies published from 2011 to 2016, the pooled HRs tended to be stable and the range of the 95% CIs became narrow in chronological order.

We also conducted a meta-regression analysis to investigate the potential source of heterogeneity among studies. However, the results showed that cancer type (*P* = 0.708), detection method (*P* = 0.144), study region (*P* = 0.358), blinding status (*P* = 0.320), sample size (*P* = 0.963), and analysis method (*P* = 0.068) did not contribute to the source of heterogeneity for OS.

### Sensitivity analysis and publication bias

**Figure 4:**
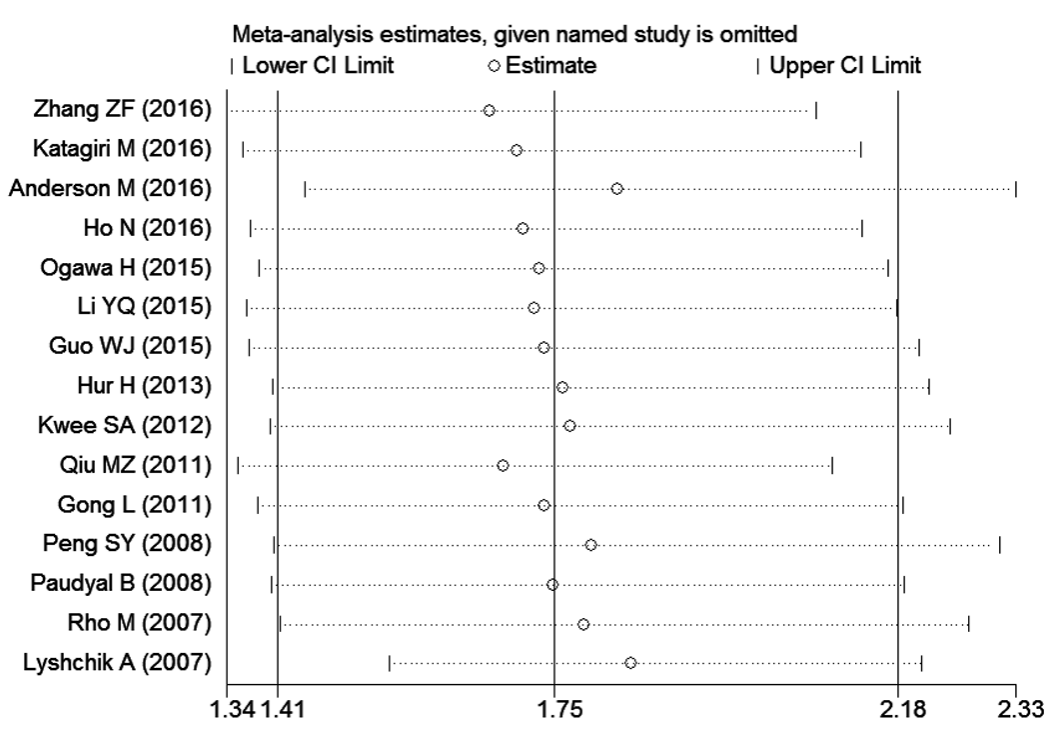
Effect of individual studies on pooled hazard ratios (HRs) for the relationship between HK2 expression and prognosis of digestive system tumors The vertical axis at 1.75 indicates the overall HR, and the two vertical axes at 1.41 and 2.18 indicate its 95% confidence interval (CI). Every hollow round indicates the pooled HR when the left study was omitted in a meta-analysis with a random model. The two ends of every broken line represent the respective 95% CI.

Sensitivity analysis suggested that no point estimate of the omitted individual dataset lay outside the 95% CI of the combined analysis based on the overall HR estimate of OS (Figure 4). These results indicated that no individual study dominated the meta-analysis results, and the outcomes were stable and reliable.

**Figure 5:**
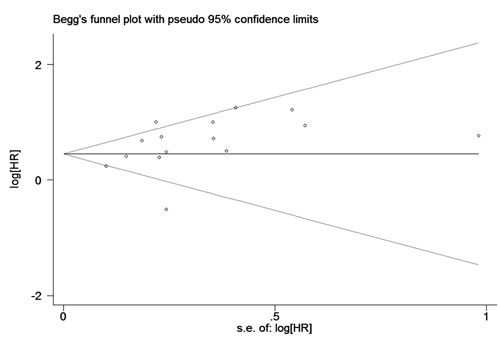
Begg's funnel plots for potential publication bias of studies reporting overall survival included in the meta-analysis Each included study represented by one circle. The horizontal line represented the pooled effect estimate.

The results of Begg's test (*P* = 0.276) and Egger’ test (*P* = 0.079) suggested that there was no statistical evidence of publication bias was found for the meta-analysis for OS. The shape of the funnel plot was symmetrical (Figure 5), which also indicated that there was no publication bias. Thus, the results of this meta-analysis were robust and reliable.

## DISCUSSION

There are three irreversible reactions in glycolysis. The first enzymatic step of glucose metabolism is catalyzed by HKs, during which glucose is ATP dependently phosphorylated to be G6P and thereby trapped within the cell. This step determines the direction and magnitude of glucose flux inside the cells, because G6P is at the branching point of several metabolic pathways, including not only glycolysis but also the pentose phosphate pathway (PPP), glycogenesis and the hexosamine pathways [31]. Thus, it would be ideal if the targeted therapy for glucose metabolism in cancer cells could be performed by cutting down glucose flux at the earliest step [32]. Remarkably, only HK2 is detected to be overexpressed in cancer cells and contributed to the high glycolytic rate in tumors. Given its selective expression in cancer, HK2 has been achieved increasing attention on its clinical implications. To date, the prognostic value of HK2 in various cancers has been extensively explored in a group of original researches. According to the first meta-analysis of HK2 overexpression related to prognosis of various solid tumors, Liu et al [33] extracted data from 21 studies and found that elevated HK2 expression was significantly associated with shorter OS and PFS. Although the prognostic value of HK2 in some cancer types of digestive system was also reported in this meta-analysis, the number of included studies was not relatively enough and at least 2 eligible studies were not included, of which one study about HCC [16], and the other about PDAC [13], were absolutely not included. So, it was hard to judge the exact impact of HK2 expression on prognosis of digestive system malignancies based on the conclusion by Liu et al. Furthermore, the current studies of HK2 expression and prognosis of various cancers were mainly focused on digestive system. In view of this, we specially conducted this meta-analysis to explore the prognostic value of HK2 on digestive system.

Our meta-analysis included 15 studies with 1932 patients, and the combined outcomes showed that high density of HK2 expression was significantly associated with worse OS in solid tumors of digestive system; hence, HK2 overexpression could be an independent unfavorable predictor of prognosis in patients with digestive system malignancies. Given that traditional meta-analysis only reflects the outcome at a certain point of time, we conducted a cumulative meta-analysis to explore the variation trend of the overall effect with the passage of time [34]. The cumulative meta-analysis revealed that the pooled HRs became stable and the range of 95% CIs became narrow as the number of studies and patients increased, which suggested that our findings were reliable. Moreover, no publication bias was found concerning the pooled outcome, and sensitivity analysis further supported the robustness of the present meta-analysis outcomes.

Despite the robustness of the pooled results, the findings should be interpreted in caution. First, the heterogeneity among the included studies was extreme in our meta-analysis, even when we conducted subgroup analyses. The significant heterogeneity could be probably caused by the differences in the patient features, cancer types, ethnicity, study protocol, and literature quality. A meta-regression analysis was performed to find out the source of heterogeneity. However, none of these confounding factors could completely explain the heterogeneity. Second, according to the results of subgroup analyses, the overall outcomes did not changed significantly based on the grouping of detection method, analysis method, sample size, blinding status, and study region. However, when grouping by tumor type, the pooled results were also statistically significant in GC, HCC, and CRC, but not in PDAC, which was similar to our previous report about the association of pyruvate kinase M2 (PKM2) expression and prognosis of digestive system cancers [34]. Since both HK2 and PKM2 are the key enzymes of glycolysis and play a critical role in promoting Warburg effect, we make a bold hypothesis that the function of HK2 may be tissue specific, and Warburg effect may represent a specific mechanism in PDAC different from other solid tumors of digestive system. However, due to the extremely different results of these three included studies and the small sample size (235 cases), our assumption was not powerfully evidenced based [18, 20, 30]. Also, the functional regulation of HK2 within various malignancies is not yet confirmed. Thus, more well-designed studies are warranted to explore the realistic prognostic effect of PKM2 on these cancers.

To further investigate the prognostic impact of HK2 on digestive system cancers, we analyzed the correlation between HK2 expression and clinicopathological factors that may affected the survival outcomes. According to the pooled results, the abnormal expression of HK2 was significantly associated with some clinical parameters, such as large tumor size, positive lymph node metastasis, advanced clinical stage, and high AFP level. All of these factors have been documented to be the powerful variables related to tumor progression and compromise long-term survival [35, 36]. Herein, high intensity of HK2 facilitates tumor progression through different pathways, which is contributed to poor prognosis in solid tumors of digestive system. In tumor cells, HK2 plays a critical role at the focus point of two central pathways of glycolysis control – c-Myc and hypoxia-inducible factor 1-alpha (HIF1α) pathways to provide tumor cells with energy and metabolic compounds for the synthesis of nucleotides and proteins [37]. HK2 interacts with the voltage-dependent anion channel (VDAC) in the outer mitochondrial membrane (OMM), and then gains both direct access to mitochondrial sources of ATP, and protection from inhibition by high level of G6P [38, 39]. The binding of HK2 to VDAC is the key event in antiapoptosis in tumor cells, which helps to decrease the formation of permeability transition pores (PTPs) in OMM and prevent subsequent release of pro-apoptotic proteins such as cytochrome *c* [40–42]. Also, the HK-VDAC complexes prevents the binding of VDAC to B-cell lymphoma-extra large (Bcl-xL) to promote Bax-Bcl-xL interactions, and subsequently blockade the mitochondrial permeabilization, resulting in the inhibition of the mitochondrial-mediated apoptotic cell death [43–46]. Therefore, the overproduction of HK2 in tumor cells provides both a metabolic benefit and an apoptosis evasive capacity, resulting in uncontrolled tumor proliferation within the host's tissues and drug resistance to chemotherapy [47]. The above evidence could partly explain the association of elevated HK2 expression with certain phenotypes of tumor progression, but the exact mechanism is not very clear and needs more investigation. Moreover, the dual role of HK2 in tumor cells makes it an attractive target for anti-cancer therapy. To date, several targeting HK2 strategies have been developed in clinic, including direct HK2 repression such as 3-bromopyruvate and lonidamine, as well as indirect HK2 suppression, such as RNA interference approaches and the abrogation of VDAC-HK complexes [48–50]. Also, digestive system cancers patients with large tumor size, positive lymph node metastasis, advanced clinical stage, and high AFP level may benefit most from HK2 evaluation to make clinical decisions.

Some limitations of our meta-analysis should be acknowledged. First, due to the unified cut-off values and follow-up times among the included studies, heterogeneity may be virtually brought in. Second, several individual HRs were calculated from survival curves or univariate analysis, which may be less reliable than the actual HRs directly obtained from published statistics [51]. Third, the differences between various protocols in HK2 detection (detection method, experimental design, specimen preparation, choice of antibody, dilution of antibodies, and other relevant information) may have confounded the pooled outcomes. Fourth, because of limited number of studies, the total sample size of Northern American population was only 344 patients, which might be not evidence-based enough, and was needed to be solved by conducting more studies with large sample. Fifth, the follow up period were extremely different among the 15 included studies, and even five studies did not report it, which may be a potential confounding factor of heterogeneity. However, due to the small number of included studies, we failed to conduct a subgroup analysis based on the follow up period. Therefore, more studies are needed to further explore the impact of this confounding factor on the pooled results. Finally, anticancer therapy has been proved to affect the survival time of cancer patients. However, whether HK2 is an independent prognostic factor from clinical treatment is still unknown because several included studies failed to control the latter.

## CONCLUSIONS

In conclusion, our meta-analysis provides evidence that HK2 may be a potential marker to predict the risk of all-caused mortality and cancer progression in patients with solid tumors of digestive system. High expression of HK2 may not only predict poor prognosis but might also be a promising therapeutic approach for developing strategies against this protein. Due to the limitations, further data are required to validate the clinical importance of HK2 by large multicenter prospective studies with larger sample sizes.

## MATERIALS AND METHODS

### Search strategy and study selection

A systematic computer-aided literature search of the PubMed, Embase, Web of Science, Cochrane Library, and China National Knowledge Infrastructure databases was conducted (last updated in January 2017) by using the following terms: “HK2 or hexokinase 2 or hexokinase II or type 2 hexokinase (all fields), cancer or tumor or malignancy or neoplasm or carcinoma (all fields), and digestive system or alimentary system (all fields), and prognosis or prognostic or survival or outcome (all fields)”. We also screened the citation lists of the relevant studies for comprehensive search. This meta-analysis was conducted according to the guidelines of the Preferred Reporting Items for Systematic Reviews and Meta-Analyses (PRISMA) [52].

Publications were recruited in this meta-analysis when they fit all of the following criteria: (1) assessing the relationship between HK2 expression and OS in patients with digestive system tumors using a cohort design; (2) detecting HK2 protein or mRNA in tumor tissue; (3) dividing the patients into two groups, namely, HK2 positive and HK2 negative, regardless of the cutoff value; (4) providing sufficient information for estimating the HRs and 95% CIs for survival outcomes in the original data; (4) being written as full papers; (5) being with the largest patient cohort among duplicated publications by the same authors or institutes. We excluded the following studies: reviews, conference abstracts, editorials, letters, basic research, or animal experiments.

### Data extraction and quality assessment

Two authors (JYW and LRH) independently reviewed and collected information from each eligible study according to the selection criteria. Any disagreement between the reviewers was resolved by consensus. Data extracted from the studies included the name of the first authors, year of publication, study region, cancer type, duration period, follow-up time, sample size, detection method, blinding status, cutoff value, number of HK2 positive, analysis method, survival outcomes, HR estimation, and quality scores. Blinding status represented that the evaluation of HK2 was blinded to the clinical outcomes. In studies where the HRs and the corresponding 95% CIs of univariate and multivariate analyses were provided, only the latter was applied to the data synthesis because it is more precise and it considers the confounding factors. In the absence of results from multivariate analysis, HR was extracted from the univariate analysis or calculated using the Kaplan–Meier survival curves [53].

The quality of included studies was assessed by NOS according to the following categories: selection, comparability, and outcome of interest. The total score of NOS ranged from 0 to 9, and we considered studies as high quality if they met at least six scores.

### Statistical analysis

STATA 11.0 software (STATA Corporation, College Station, TX, USA) was used for all statistical analysis. The combined HR and 95% CI were used to assess the strength of HK2 expression with survival endpoints (OS) based on the data extracted from the eligible studies. HR > 1 indicated an increased risk of poor prognosis for patients with HK2 overexpression when the 95% CI exceeding 1. The statistical significance of the pooled HR was determined through Z–test. The results were considered statistically significant if *P* < 0.05. Subgroup analyses were conducted according to cancer type (at least two trials must report the same outcome for the same cancer type; otherwise, they will be assigned to a subgroup designated “Others”), detection method (“IHC”, “RT-PCR”, and “IF”), study region (“Eastern Asia” and “America”), blinding status (“yes” and “none reported”), and sample size (“≥ 100” and “< 100”). Meta-regression analysis was also performed to determine the potential sources of heterogeneity. For the pooled analysis of the correlation between HK2 expression and clinicopathological features (i. e., gender, tumor size, depth of invasion, lymph node metastasis, clinical stage, differentiation, distant metastasis, AFP level, HBV infection, liver cirrhosis, and portal vein involvement), the ORs and their corresponding 95% CI were combined to estimate the effect. All statistical tests were two sided.

Heterogeneity assumption was qualitatively examined through the chi-squared test based on the *Q* statistic, and was considered statistically significant when *P* < 0.05. Heterogeneity was also quantitatively estimated using the *I*^2^ metric (*I*^2^ < 25%, no heterogeneity; *I*^2^ = 25% – 50%, moderate heterogeneity; *I*^2^ > 50%, extreme heterogeneity) [54]. When significant heterogeneity had been observed among the studies (*P* < 0.05 or *I*^2^ > 50%), the pooled HR estimation of each study was calculated using a random-effects model (DerSimonian and Laird method). Otherwise, a fixed-effects model was applied (Mantel–Haenszel method) [55]. Sensitivity analysis was conducted by sequentially removing each individual study to validate the stability of the pooled outcomes. Publication bias was statistically assessed by Begg's and Egger's asymmetry tests (*P* < 0.05 was defined as statistically significance) [56], and was visually evaluated using funnel plots.

## SUPPLEMENTARY MATERIALS FIGURES AND TABLES




