# Supplementary material for: Poor prognosis of hexokinase 2 overexpression in solid tumors of digestive system: a meta-analysis
Source: Oncotarget. 2017 Mar 7;8(19):32332–44. doi: 10.18632/oncotarget.15974 (PMC5458288; doi:10.18632/oncotarget.15974)
Supplement: Supplementary file 1 [file oncotarget-08-32332-s001.pdf]

Poor prognosis of hexokinase 2 overexpression in solid tumors of digestive system: a meta-analysis

Supplementary Material

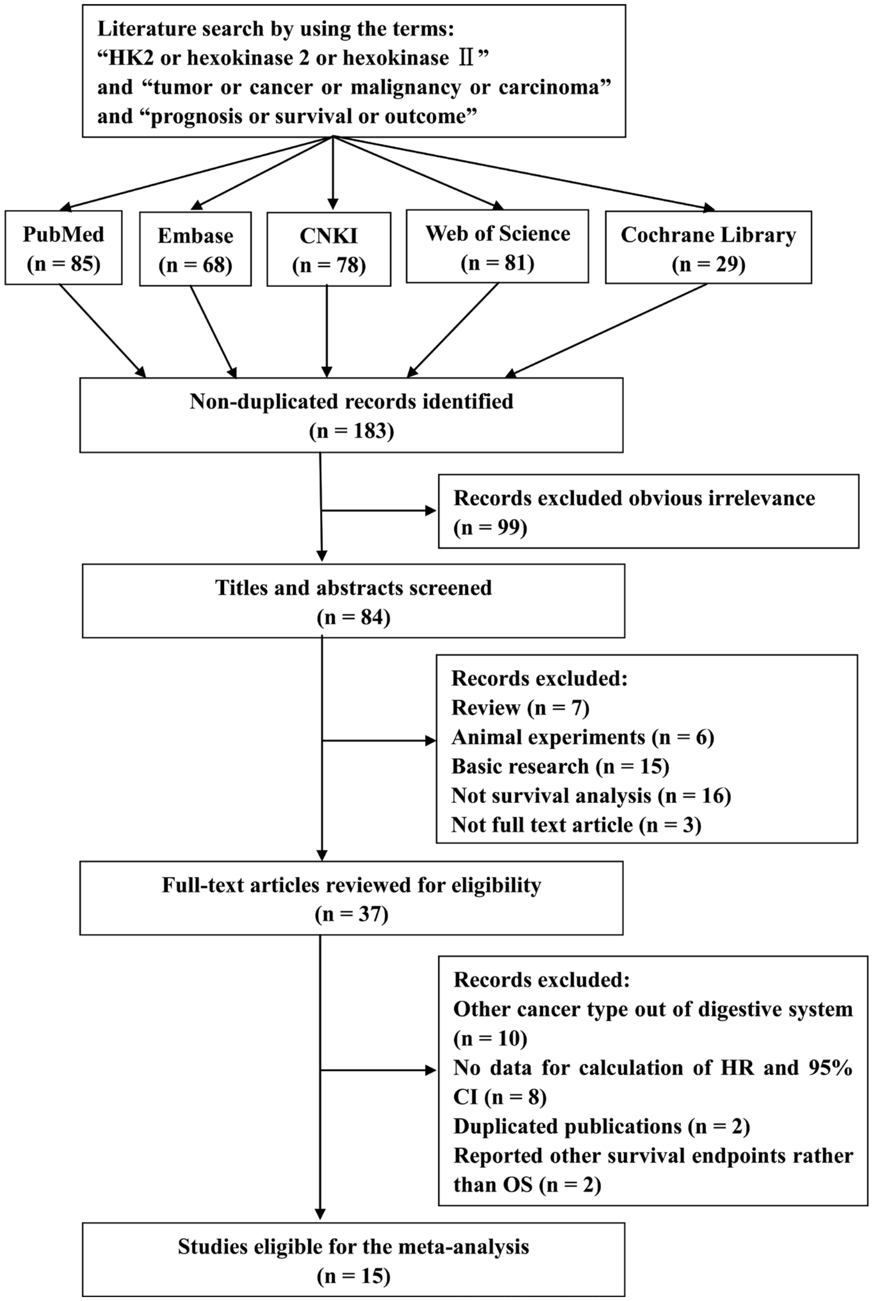

Figure S1: The complete flow diagram of the electronic literature search process. By using the searching terms: "HK2 or hexokinase 2 or hexokinase II" and "tumor or cancer or malignancy or carcinoma" and "prognosis or survival or outcome", a plenty of studies were preretrieved from the basic datasets, including 85 from PubMed, 68 from Embase, 78 from CNKI, 81 from Web of Science, and 29 from Cochrane Library. 183 non-duplicated records were identified. Of these articles, 99 were excluded because of clear lack of relevance. The remaining 84 studies were further screened out through browsing the titles and abstracts, and then 47 were removed based on the eligible criteria. After reading the full texts of 37 studies, 15 eligible studies were finally included in this meta-analysis.
